# Treatment of a Developmental Groove and Supernumerary Root Using Guided Tissue Regeneration Technique

**DOI:** 10.1155/2016/2738569

**Published:** 2016-11-14

**Authors:** Zahra Alizadeh Tabari, Hamed Homayouni, Tahere Pourseyediyan, Armita Arvin, Derrick Eiland, Nima Moradi Majd

**Affiliations:** ^1^Department of Periodontics, Dental School, Qazvin University of Medical Sciences, Qazvin 34157-59811, Iran; ^2^Department of Endodontics, Dental School, Qazvin University of Medical Sciences, Qazvin 34157-59811, Iran; ^3^Department of Endodontics, Dental School, Yazd University of Medical Sciences, Yazd 8914881167, Iran; ^4^Dental Emergency Department, Howard University College of Dentistry, Washington, DC 20001, USA; ^5^Dental Research Laboratory, Howard University College of Dentistry, Washington, DC 20001, USA

## Abstract

*Introduction*. The radicular groove is a developmental groove which is usually found on the palatal or lateral aspects of the maxillary incisor teeth. The present case is a maxillary lateral incisor with a small second root and a deep radicular groove. The developmental groove caused a combined periodontal-endodontic lesion.* Methods*. Case was managed using a combined treatment procedure involving nonsurgical root canal therapy and surgical periodontal treatment. After completion of root canal treatment, guided tissue regeneration (GTR) was carried out using decalcified freeze dried bone allograft (DFDBA) and a bioabsorbable collagenous membrane. Tooth also was splinted for two months.* Results*. After 12 months the tooth was asymptomatic. The periapical radiolucency disappeared and probing depth did not exceed 3 mm.* Conclusion*. Combined treatment procedure involving nonsurgical root canal therapy and surgical periodontal regenerative treatment can be a predictable technique in treating combined endodontic-periodontal lesions caused by radicular groove.

## 1. Introduction

The radicular groove is a developmental groove which is usually found on the palatal or lateral aspects of the maxillary incisor teeth. It is a linear depression that sometimes reaches the apex [1]. The groove is a rare anomaly with prevalence rate of 2.8–8.5% and generally presents on the maxillary lateral incisors [2].

Radicular grooves have been classified into three groups based on their severity. Type I, the groove is limited to the coronal third of the root. Type II, the groove is extended beyond the coronal third of the root but it is shallow and pulp is not exposed. Type III, the groove is long and deep, it is extended beyond the coronal third of the root, and root canal system is involved [3].

It has been stated that the radicular groove is analogous to the pathogenesis of dens invaginatus because it happens due to a slight in-folding of the enamel organ and Hertwig's epithelial root sheath cells during odontogenesis [4].

Radicular groove can be an appropriate habitat for microorganisms and plaque accumulation. Therefore, a local inflammation and deep periodontal pocket are general findings. A deep pocket that reaches to the root apex can affect the pulp vitality and causes a combined periodontal-endodontic lesion [5].

In order to treat a radicular groove the following procedures have been suggested: curettage of the affected periodontal tissues [6], sealing the groove with a biocompatible material [7], saucerization of the groove [7], root canal therapy when an endodontic lesion is present [8], and surgical techniques (i.e., guided tissue regeneration therapy and intentional replantation) [9, 10].

Although most anatomical studies have shown that maxillary incisors always have a single root, there are case reports suggesting lateral incisor with two [11, 12] and three root canals [13]. The term “supernumerary root” has been used to describe the development of increased number of roots on a tooth compared with the classical description in dental anatomy [14].

The aim of the present paper is to report a case involving a maxillary lateral incisor with a small second root and a Type III deep radicular groove which caused a combined periodontal-endodontic lesion. Therefore, a combined treatment procedure involving nonsurgical root canal therapy and surgical periodontal treatment was required.

## 2. Case Report

**Figure 1:**
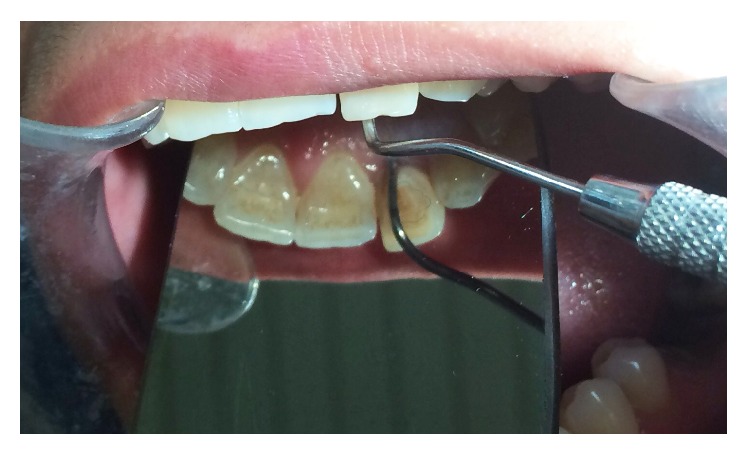
Preoperative photograph; a 7 mm localized pocket was detected on the palatal of tooth #10.

**Figure 2:**
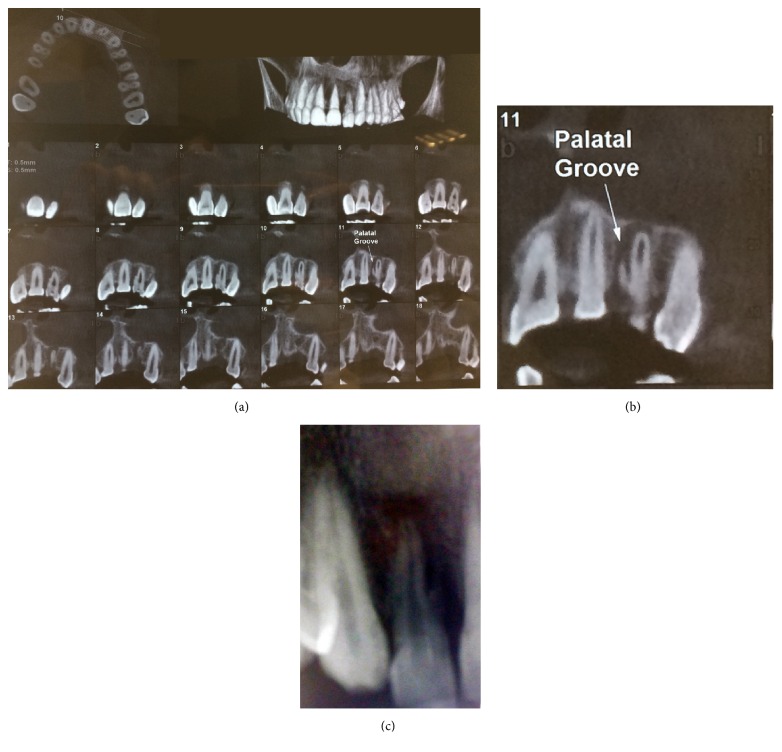
(a) Preoperative cone beam computed tomography (CBCT); (b) CBCT revealed a radicular groove on tooth #10; (c) preoperative periapical radiograph, there is a large radiolucency in relation to left lateral incisor.

A 32-year-old female patient presented to endodontic department of Qazvin School of Dentistry with a chief complaint of mobility of maxillary left lateral incisor. Clinical examination was performed. A 7 mm localized pocket was detected on the palatal of tooth #10 (Figure 1). Probing depth of the mesial, distal, and buccal gingival sulcus was within normal limits. Tooth #10 did not respond to electric pulp test (Analytic Technology, Redmond, WA, USA), cold test (Roeko Endo-Frost; Roeko, Langenau, Germany), percussion, and palpation; it also had grade I mobility. Periapical radiograph and cone beam computed tomography (CBCT) were taken (Figure 2). CBCT revealed a radicular groove on tooth #10 and a large radiolucency in relation to left lateral incisor. A supernumerary root was also observed in the radiographs.

Considering patient's history, clinical and radiographic examination, the lesion was provisionally diagnosed as necrotic pulp and localized periodontitis secondary to the radicular groove.

Treatment options were presented to the patient including a combined procedure involving endodontic and periodontal regenerative treatments. She was informed that due to length and depth of the radicular groove, long-term prognosis of tooth #10 is questionable. A written informed consent was obtained and the patient was scheduled for endodontic treatment.

## 3. Endodontic Treatment

**Figure 3:**
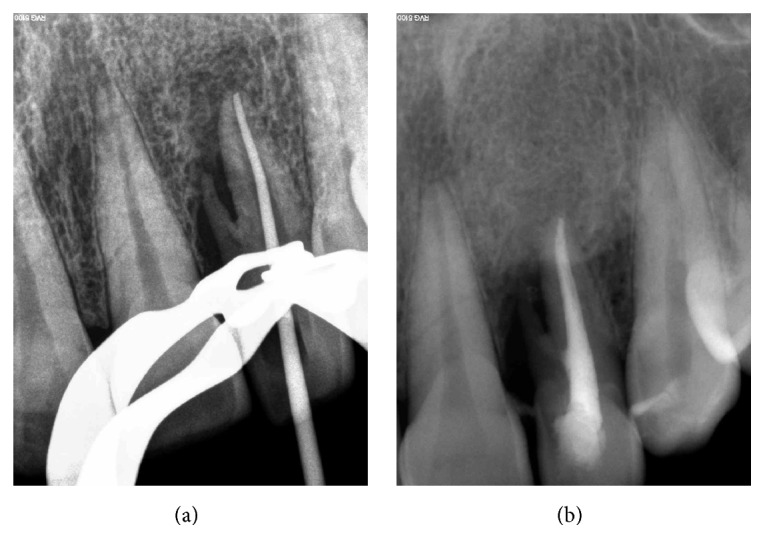
Toot #10 was endodontically treated.

At the patient's return, local anesthesia was administered using local infiltration (supraperiosteal) technique with a cartridge of lidocaine (2% lidocaine with 1/80000 epinephrine; Darupakhsh, Tehran, Iran); after proper isolation, access cavity on tooth #10 was prepared. Canal preparation was performed using the ProTaper system (Dentsply Maillefer, Ballaigues, Switzerland) according to the manufacturer's instructions. An attempt was made to negotiate the second root canal, but no canal opening was found for the supernumerary root. Sodium hypochlorite 2.5% (Kimia Tehran Acid, Tehran, Iran) was used as irrigating solution. Canal was obturated using lateral compaction technique with gutta-percha (Gapadent Co., LTD, Korea) and AH26 sealer (DeTrey, Dentsply, Konstanz, Germany). The access cavity was sealed with Cavit (Coltosol, AriaDent, Tehran, Iran) (Figure 3).

## 4. Surgical Procedure

**Figure 4:**
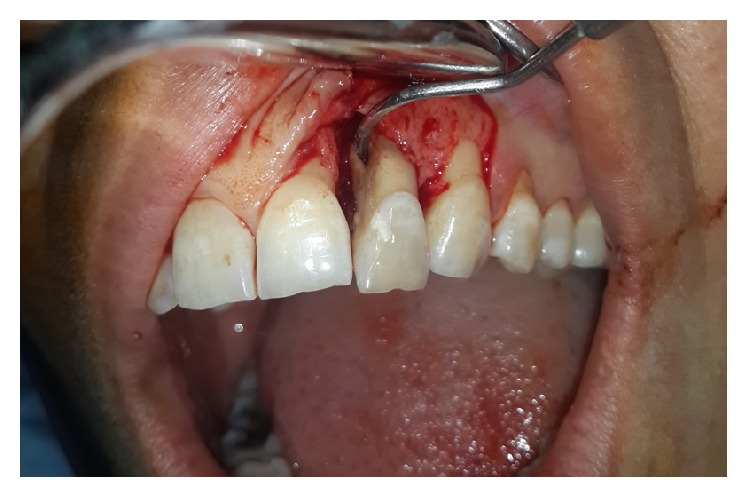
A full thickness mucoperiosteal flap was raised on labial and palatal aspects of tooth #10 to access the radicular groove.

**Figure 5:**
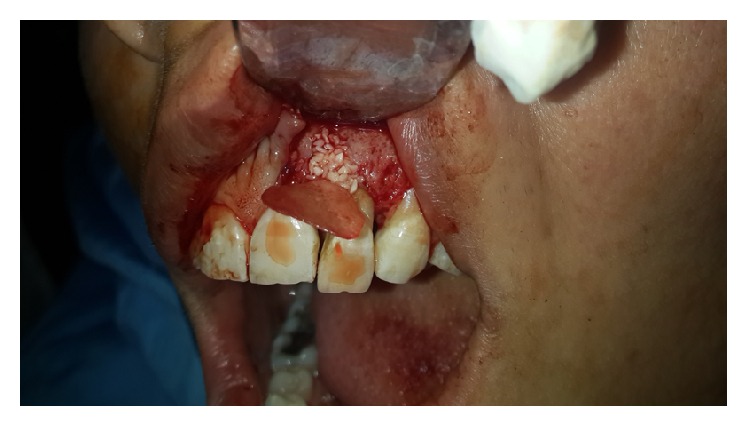
Guided tissue regeneration (GTR) was carried out using decalcified freeze dried bone allograft (DFDBA) and a bioabsorbable collagenous membrane.

The surgical area was made aseptic and local anesthesia was administered (2% lidocaine with 1/80000 epinephrine; Darupakhsh, Tehran, Iran). Sulcular incisions were placed on the labial side from #9 to #11; to increase the access a releasing incision was also made on the distal of #11. A full thickness mucoperiosteal flap was raised on labial and palatal aspects to access the radicular groove (Figure 4). Granulation tissues were removed using curettage and root planning was carried out; then the supernumerary root was cut off and groove was saucerized. No canal opening was found on the area of removed root. The supernumerary root and surrounding tissue were sent to the lab for histologic examination. Then, guided tissue regeneration (GTR) was carried out using decalcified freeze dried bone allograft (DFDBA) with particle size of 500 to 1000 *μ*m (Cenobone, Tissue Regeneration Corporation, Kish Island, Iran) and a 20 × 25 mm (0.4 to 0.6 mm thickness) bioabsorbable collagenous membrane (Cenomembrane, Tissue Regeneration Corporation, Kish Island, Iran) (Figure 5). The flap was repositioned and stabilized with sutures. The tooth was restored using composite resin and immobilized with a semirigid splint. The splint was initially placed on the buccal aspect of the tooth, but due to esthetic consideration, after four weeks, it was removed and placed lingually. The patient was prescribed a chlorhexidine gluconate mouth rinse and 4 × 400 mg Ibuprofen plus 3 × 500 mg amoxicillin daily for a week. Sutures were removed two weeks after the surgery, but the splint was remained for two months. No mobility was detected after splint removal.

**Figure 6:**
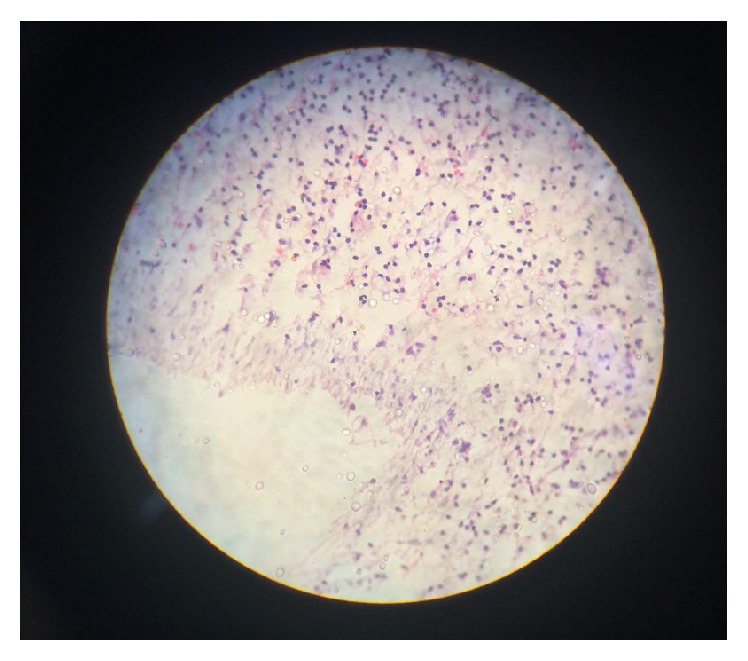
The histologic examination results revealed that the surrounding tissue consists of connective tissue and inflammatory cells and root structure is normal.

The histologic examination results revealed that the structure of supernumerary root has no abnormality, no dysplastic cell is detected, and the surrounding tissue consists of connective tissue and inflammatory cells (Figure 6).

**Figure 7:**
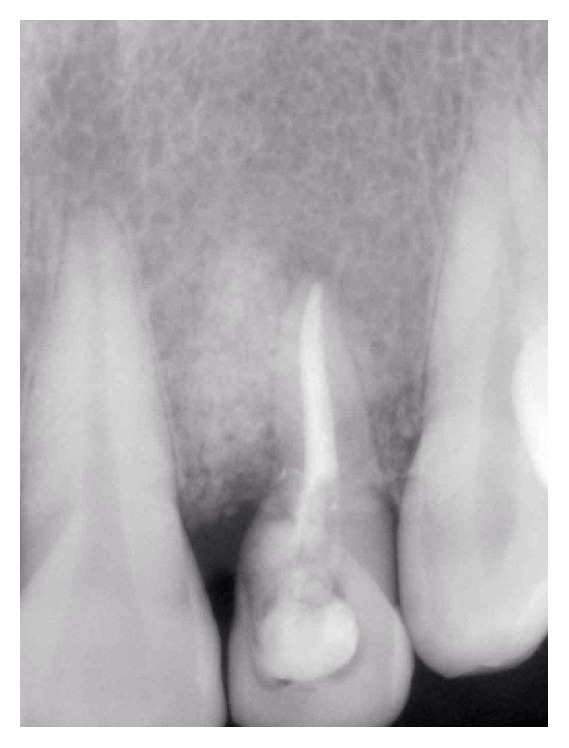
12 months' follow-up radiograph indicated disappearance of the radiolucency around the tooth #10 due to bone grafting and simultaneous bone regeneration.

Twelve months' follow-up revealed absence of signs and symptoms, and probing depth did not exceed 3 mm and radiographic examination indicated disappearance of the radiolucency around the tooth #10 due to bone grafting and simultaneous bone regeneration (Figure 7).

## 5. Discussion

Combined endodontic-periodontal lesions are real clinical dilemmas due to difficulty of making a differential diagnosis and deciding a prognosis. One of the serious factors that causes this kind of combined lesions is radicular groove. These developmental grooves act as “plaque trap” and initiating factor in localized gingivitis and periodontitis [15]. In cases with more complicated grooves (Type III), focal attachment loss may extend apically and result in a hopeless periodontal prognosis. It has been stated that long lasting deep periodontal pocket can secondarily lead to pulp necrosis and develop a combined endodontic-periodontal lesion [16].

The key factor to achieve success in management of this type of anomalies is accurate diagnosis [17]. Therefore, to obtain a three-dimensional image of the tooth and to determine its accurate prognosis, a CBCT image was taken.

Kerezoudis et al. [18] suggested the following treatment modalities to manage radicular grooves:

1. Surgical removal of granulation tissue and irritants
2. Gingivectomy and apically positioned flap
3. Surgical exposure and flattening of the groove by grinding, with or without application of guided tissue regeneration techniques
4. Placing amalgam restoration in the groove
5. Orthodontic extrusion of the tooth

Although shallow grooves which are located entirely on the crown can be corrected by odontoplasty and curettage of granulation tissue, more complicated radicular grooves that are associated with severe periodontal breakdown and extensive periapical lesion need surgical intervention [19].

In our case, due to presence of deep periodontal pocket and severe attachment loss, surgical procedure was performed. After flattering and odontoplasty of the groove, the anatomy of the root was favorable. That is why no restorative material was used to restore the groove.

Regenerative periodontal therapy aims to predictably restore the periodontal tissue and leads to formation of a new cementum with inserting periodontal ligament fibers [20].

Therefore, to enhance the periodontal attachment of the tooth in the present case, DFDBA was used. It has been stated that DFDBA is a scaffold for bone formation and is able to be converted into bone [21]. Also, to provide epithelial exclusion and allow periodontal ligament, cementum, and bone to regenerate [22], a collagenous membrane was placed over the defect.

The applied treatment regimen was successful as it can be observed on the follow-up radiography. After 12 months the subjected tooth was asymptomatic, and a 3 mm healthy gingival sulcus was restored in relation to the radicular groove.

## 6. Conclusion

Combined treatment procedure involving nonsurgical root canal therapy and surgical periodontal regenerative treatment can be a predictable technique in treating combined endodontic-periodontal lesions caused by radicular groove.
